# Exploring Inflammatory Parameters in Lung Cancer Patients: A Retrospective Analysis

**DOI:** 10.3390/jpm14060552

**Published:** 2024-05-22

**Authors:** Catalin Vladut Ionut Feier, Calin Muntean, Alaviana Monique Faur, Vasile Gaborean, Ioan Adrian Petrache, Gabriel Veniamin Cozma

**Affiliations:** 1 First Discipline of Surgery, Department X-Surgery, “Victor Babes” University of Medicine and Pharmacy, 2 E. Murgu Sq., 300041 Timisoara, Romania; catalin.feier@umft.ro; 2 First Surgery Clinic, “Pius Brinzeu” Clinical Emergency Hospital, 300723 Timisoara, Romania; 3 Medical Informatics and Biostatistics, Department III-Functional Sciences, “Victor Babes” University of Medicine and Pharmacy, 2 E. Murgu Sq., 300041 Timisoara, Romania; 4 Faculty of Medicine, “Victor Babes” University of Medicine and Pharmacy, 300041 Timisoara, Romania; alaviana.faur@student.umft.ro; 5 Thoracic Surgery Research Center, “Victor Babeş” University of Medicine and Pharmacy Timisoara, Eftimie Murgu Square No. 2, 300041 Timisoara, Romania; vasile.gaborean@umft.ro (V.G.); ioan.petrache@umft.ro (I.A.P.); gabriel.cozma@umft.ro (G.V.C.); 6 Department of Surgical Semiology, Faculty of Medicine, “Victor Babeş” University of Medicine and Pharmacy Timisoara, Eftimie Murgu Square No. 2, 300041 Timisoara, Romania

**Keywords:** lung cancer, inflammation biomarkers, smoking, air leaks, systemic immune-inflammation index, prognosis

## Abstract

Inflammation-related parameters serve as pivotal indicators in the prognosis and management of lung cancer. This retrospective investigation aimed to explore the relationship between inflammatory markers and diverse clinical variables in non-small-cell lung cancer patients. A cohort of 187 individuals undergoing elective lobectomy for lung cancer was retrospectively analyzed, spanning an 11-year data collection period. Six inflammation ratios derived from complete peripheral blood counts were assessed. Significantly elevated levels of neutrophil-to-lymphocyte ratio (NLR) (*p* = 0.005), platelet-to-lymphocyte ratio (PLR) (*p* = 0.001), Aggregate Index of Systemic Inflammation (AISI) (*p* = 0.015), Systemic Inflammation Response Index (SIRI) (*p* = 0.004), and Systemic Immune Inflammation Index (SII) (*p* = 0.004) were observed in patients with advanced T stages. Significantly, elevated values (*p* < 0.05) of these parameters were observed in the study’s smoker patients compared to non-smokers. A statistically significant correlation was identified between the NLR parameter and tumor size (*p* = 0.07, r = 0.204), alongside a significant elevation in SIRI (*p* = 0.041) among patients experiencing postoperative complications. Inflammatory biomarkers emerge as invaluable prognostic indicators for patients with non-small-cell lung cancer, offering potential utility in forecasting their prognosis.

## 1. Introduction

Lung cancer is a global public health problem, holding a leading position in terms of incidence and mortality in both sexes. This disease represents a burden for the Romanian medical system, with this country being no exception to the rule, through the obscure prognosis suggested by the highest mortality among all cancers, registering over 10,500 deaths in 2022, according to Globocan [1]. Moreover, cancers of the respiratory tract seem to be following an ascending pattern in terms of both incidence and mortality in both sexes worldwide until 2050 as per the Global Cancer Observatory-Cancer Tomorrow (https://gco.iarc.who.int/en (accessed on 22 April 2024)) [2], a fact that justifies the need to explore new effective parameters in determining the evolution of this pathology.

Non-small-cell lung cancer (NSCLC) treatment encompasses various therapeutic approaches, including surgery, radiotherapy, chemotherapy, and targeted therapies, each with a distinct impact on tumor inflammation and cellular infiltration [3]. Surgical resection reduces tumor mass and decreases the inflammatory burden, potentially enhancing immune response [4,5]. Radiotherapy induces an inflammatory response that recruits immune cells, possibly boosting anti-tumor immunity but also causing collateral tissue damage [4,6]. Chemotherapy modifies the tumor microenvironment by reducing tumor-associated inflammatory cells, potentially hindering tumor progression [4,7].

Inflammatory cell infiltration in the tumor microenvironment includes various cells such as neutrophils, macrophages, and lymphocytes, each contributing to tumor progression through distinct mechanisms. Tumor-associated neutrophils (TANs) and tumor-associated macrophages (TAMs) are crucial components influencing cancer progression by promoting tumor growth, angiogenesis, genetic instability, and cellular invasiveness. For instance, TANs, derived from peripheral neutrophils, can stimulate angiogenesis and cell proliferation by releasing pro-angiogenic factors and proteolytic enzymes. Conversely, TAMs, originating from circulating monocyte precursors, contribute to tumor progression by secreting angiogenic factors and enzymes that degrade the extracellular matrix [8,9].

Therefore, our study aims to evaluate whether increased preoperative values of systemic inflammatory markers in patients undergoing lung cancer surgery correlate with a higher incidence of postoperative complications and, implicitly, with a worse prognosis.

Systemic inflammatory markers are easy to determine preoperatively and represent a cost-effective modality for the insights they provide. They have the potential to estimate the progression of cancer, especially regarding the short-term prognosis, correlated with the incidence and severity of post-procedural complications, but also the long-term evolution, by predicting the recurrence rate of the disease and overall survival [10,11].

Regarding lung cancer, various inflammatory biomarkers were analyzed preoperatively and proved usefulness in estimating the postoperative evolution from several points of view: neutrophil-to-lymphocyte ratio (NLR) and platelet-to-lymphocyte ratio (PLR) correlate with the incidence and severity of postoperative complications [12]. Systemic Inflammation Response Index (SIRI) and Aggregate Index of Systemic Inflammation (AISI) may help identify patients who will require more postoperative hospital days and will encounter a worse prognosis. Some studies have shown the clinical value of systemic markers with predilection in the early stages of lung cancer; higher NLR (>=2.606), SIRI (>=0.705), and SII (>=580.67) bear a poor prognosis of overall survival, with increased relevance in stage I therapy [13]. Lymphocyte-to-monocyte ratio (LMR) and PLR disclose statistical significance in determining long-term survival in stage IB NSCLC [14]. LMR is considered an accurate predictor of disease recurrence in stage IA NSCLC [10].

Although various studies point out the independent value of certain inflammatory biomarkers to predict overall survival [15,16,17,18], some state that combined scoring could have a higher predictive potential concerning the prognosis [19,20].

This study presents data from patients who underwent surgery for lung cancer during a period of 11 years at the Thoracic Surgery Clinic of the Municipal Clinical Hospital in Timisoara, Romania. The study aims to assess how preoperative inflammatory markers influences the postoperative prognosis of patients undergoing lobectomy for NSCLC, including adenocarcinomas and squamous cell carcinomas. It also aims to evaluate the degree of association between systemic biomarkers and predetermined behaviors or characteristics of patients, such as smoking, but also the individual ability of each marker to link to the postoperative status.

## 2. Materials and Methods

To conduct this retrospective study, data from 187 patients who underwent open elective surgery for lung cancer, specifically lobectomy, were analyzed. This retrospective study encompassed data from patients who underwent surgery at the Thoracic Surgery Clinic of the Municipal Emergency Clinical Hospital in Timisoara over an 11-year period, from 1 January 2013 to 31 December 2023.

### 2.1. Criteria for Participant Selection in the Study

Inclusion and exclusion criteria were established for this study.

Inclusion Criteria:

- Patients undergoing surgical treatment for lung cancer.
- Patients with histopathological diagnoses of either adenocarcinoma or squamous cell carcinoma, categorized as subtypes of NSCLC.
- Tumor limited to a single lobe, followed by lobectomy.
- Patients undergoing surgical treatment during the specified timeframe

Additionally, exclusion criteria were developed. Due to the impact of chemotherapy and radiotherapy on the inflammatory system [21,22], patients who underwent neoadjuvant oncologic treatment were excluded.

Data from patients presenting with pulmonary metastases from other cancer types or those in stage IV of lung cancer were not considered.

Considering that this extended period included an unprecedented crisis in recent history, namely the COVID-19 pandemic, patients undergoing surgical treatment during this period were required to meet additional inclusion criteria. Due to the significant impact of SARS-CoV-2 infection on the inflammatory status of patients [23,24], patients with a history of positive COVID-19 prior to hospitalization or who developed this infection during hospitalization were excluded. Moreover, the presence of specific symptoms of this pathology upon admission or within 7 days prior to admission was considered an exclusion criterion. Lastly, during the pandemic, only patients with a negative RT-PCR test upon admission to the clinic or within 24 h prior to admission were considered.

### 2.2. Data Collection and Prognostic Evaluation Parameters

Once the inclusion criteria were met, data collection proceeded for the purpose of analysis and statistical interpretation. Subsequently, after assessing demographic aspects (gender, age, rural/urban origin), the collection of leukocyte formula values was conducted as follows:

- Lymphocyte (Lym);
- Monocyte (Mon);
- Neutrophil (Neu);
- Platelet (Pla).

After obtaining these data, the subsequent step involved calculating various parameters of inflammatory status, as follows:

- LMR (lymphocyte/monocyte ratio) = Lym/Mon;
- NLR (neutrophil/lymphocyte ratio) = Neu/Lym;
- PLR (platelet/lymphocyte ratio) = Pla/Lym;
- SII (Systemic Immune Inflammation Index) = (Neu × Pla)/Lym;
- AISI (Aggregate Index of Systemic Inflammation) = (Neu × Mon × Pla)/Lym;
- SIRI (Systemic Inflammation Response Index) = (Mon × Pla)/Lym.

To assess the prognostic evaluation capacity of these parameters, associations with other study variables were elucidated. Specifically, consideration was given to patients’ smoking status. Given the broad spectrum of comorbidities they presented, the Charlson index was employed to provide an overview of the breadth of associated pathologies [25]. Tumor location, along with the extent of tumor invasion (T), lymph node involvement (N), and absence of metastases (M), were additional parameters considered. Correlating with TNM staging, the patient’s disease stage and tumor size were highlighted. Regarding postoperative progression, the occurrence of air leaks was analyzed as a postoperative complication, along with the number of days patients spent in intensive care units (ICUs). Lastly, an analysis encompassing total hospitalization duration, as well as pre- and postoperative hospitalization durations, alongside surgical intervention duration, was conducted.

This study was conducted according to the Declaration of Helsinki. The study received approval from the Ethical Commission of Municipal Emergency Clinical Hospital in Timisoara (No.E-2635/26 April 2024).

### 2.3. Statistical Analysis

Following the collection of all data, statistical analysis was conducted using IBM SPSS Statistics 25 software for Windows (IBM, Armonk, NY, USA). Measures of central tendency and dispersion were computed for numerical variables, and statistical differences between two samples were assessed using the Mann–Whitney test. For comparison among three or more sample groups, ANOVA testing was applied. Regarding categorical variables, frequency tables and percentages were utilized for evaluation. Pearson correlation coefficient (r) was employed for analyzing correlations between variables, while the chi-squared test was applied for comparing proportions across different sample groups. All obtained results were deemed statistically significant at a level of *p* < 0.05.

## 3. Results

### 3.1. Key Information

The data of 187 patients who met the inclusion criteria for this study were analyzed. Their ages ranged from 22 to 83 years, with a mean (M) of 60.43 years and a standard deviation (SD) of 9.03 years. The 11-year period also encompassed the COVID-19 pandemic (from 26 February 2020, the date of the first confirmed COVID-19 case in Romania, to 8 March 2022, when all imposed restrictions were lifted), during which only 19 surgical interventions were performed. The general characteristics of the patients participating in the study are presented in Table 1.

Male patients exhibited a mean total hospitalization duration of 15.76 ± 7.45 days, while female patients showed a mean total hospitalization duration of 13.63 ± 5.51 days. Statistical tests for detecting differences yielded a *p*-value of 0.039 between the two. Additionally, the surgical intervention duration was 277.85 ± 73.3 min for male patients and 243.85 ± 64.83 min for female patients, with statistical tests revealing a *p*-value of 0.012.

The mean value of the Charlson comorbidity index was 4.97 ± 1.46 for the entire cohort. Smoker patients had an average index of 4.88 ± 1.25, whereas non-smokers had a mean index of 5.02 ± 1.56. Upon conducting statistical tests to highlight the results between the two groups, a *p*-value of 0.488 was obtained. Additionally, a significant positive correlation was observed between this index and NLR (*p* = 0.042, r = 0.149).

Patients with a histopathological type of squamous cell carcinoma required a mean intervention duration of 279.6 ± 66.97 min, while those with adenocarcinoma had a mean duration of 251.62 ± 72.44 min. Statistical tests yielded a *p*-value of 0.044. Additionally, in patients with squamous cell carcinoma, the mean neutrophil count was significantly higher compared to those with adenocarcinoma (6203 ± 2342 vs. 5257 ± 2136, *p* = 0.01). Furthermore, a significant increase in the AISI marker (613.49 ± 650.24 vs. 427.6 ± 420.71, *p* = 0.031) was observed in these patients.

The variation in inflammatory parameters in smokers compared to non-smokers is presented in the Table 2.

The size of excised tumors ranged from 0.3 cm to 10.5 cm. Further statistical analysis revealed a statistically significant correlation between this parameter and NLR (*p* = 0.07, r = 0.204).

### 3.2. Stage of Disease and Outcomes

There were no differences in the mean age of patients based on disease stage (61.62 ± 9.52 vs. 59.65 ± 8.8 vs. 60.59 ± 8.99, *p* = 0.432).

Male patients presented a subgroup of stage II or III in 76 cases (40.64%), compared to 31 cases (15.57%) in female patients. After applying the chi-squared test to highlight differences in proportions, a *p*-value of 0.039 was obtained. Furthermore, analyzing the variation in mean AISI between the two genders yielded a *p*-value of 0.001. The variation in inflammatory parameters based on disease stage is depicted in the Table 3.

Patients spent an average of 2.59 ± 1.947 days in ICUs. Smokers required an average ICU monitoring period of 2.83 ± 2.46 days, compared to 2.45 ± 1.59 days for non-smokers. Statistical tests for detecting differences between the two groups yielded a *p*-value of 0.263.

Analyzing T stage, no significant differences were observed in the number of lymphocytes, monocytes, platelets, or neutrophils between patients presenting with tumor invasion stages T1-T2 and T3-T4.

However, there were significant differences between T1-T2 and. T3-T4 regarding the biomarkers investigated. The results are presented in Table 4.

There were no significant differences observed in the ages of patients who experienced air leaks as a postoperative complication (61.44 ± 8.38 vs. 59.94 ± 9.71, *p* = 0.301). Patients who experienced air leaks spent an average of 3.53 ± 1.97 days in ICUs compared to 2.12 ± 0.89 days for those who did not. Statistical analysis was conducted to highlight differences between the means of the two patient categories that resulted in *p* < 0.001. The same trend was evident in the analysis of total hospitalization duration (17.73 ± 8.29 days vs. 13.58 ± 5.54 days, *p* < 0.001) and postoperative hospitalization duration (14.18 ± 7.26 days vs. 9.62 ± 4.17 days, *p* < 0.001).

The differences between patients with and without air leaks, regarding the biomarkers, are presented in Table 5.

Patients with air leaks were approximately equally distributed between rural and urban environments (30 (48.8%) vs. 32 (51.6%)). However, among patients who did not experience air leaks, 84 (72.4%) were from urban areas compared to only 41 (32.8%) from rural areas. Applying the chi-squared test to highlight differences in proportions among those without complications yielded a *p*-value of 0.039.

## 4. Discussion

Over time, numerous studies have demonstrated the relationship between various types of cancer and inflammatory status [14,26,27]. Lung cancer is no exception to this rule, with evaluated inflammatory markers proving significantly valuable in assessing patient prognosis and the occurrence of postoperative complications [18,28]. With their cost-effectiveness and routine preoperative availability, individual analysis of the leukocyte formula markers can be easily determined, and the relevance of their variation becomes increasingly significant [19].

Tumor-associated neutrophils (TANs) and tumor-associated macrophages (TAMs) are vital components of the tumor microenvironment, influencing tumor progression through various mechanisms [8]. TANs, derived from peripheral neutrophils, promote tumor growth, angiogenesis, genetic instability, and invasiveness, while TAMs, originating from circulating monocytes precursors, facilitate tumor progression by secreting angiogenic factors and protease enzymes that degrade extracellular matrices, promoting angiogenesis and tumor cell proliferation [8,9].

Conversely, lymphocytes play a crucial role in host cell-mediated immunity, aiding in the destruction of malignant cells and micrometastases. Tumor-infiltrating lymphocytes (TILs) have been associated with improved clinical outcomes in several cancers. Platelets also contribute to tumor angiogenesis by releasing angiogenesis stimulators upon adhering to tumor vessels [29,30].

This study, conducted at a Thoracic Surgery Clinic on a cohort of 187 patients, aimed to analyze various inflammatory parameters (LMR, NLR, PLR, AISI, SIRI, SII) derived from leukocyte formula components and establish their preoperative variation based on various evaluated parameters.

In the initial phase, the patient cohort was stratified into smokers and non-smokers undergoing surgery for lung cancer. Significant differences were observed regarding the average age of smokers, who were notably younger (*p* = 0.025), and predominantly male (*p* = 0.026). Furthermore, these patients experienced air leaks as a postoperative complication in a significantly higher proportion (*p* = 0.047). Male patients exhibited a higher proportion of stage II or III disease (*p* = 0.039) and had a higher mean AISI value than females (*p* = 0.001).

It is well established that tobacco consumption is the primary factor in the onset and progression of lung cancer, with a decrease in tobacco use inevitably leading to a reduction in disease incidence [31]. The increased incidence of lung cancer among men, supported by this study, is primarily attributed to higher tobacco consumption among males [31,32,33]. Jeganathan et al. illustrate that smokers are more prone to developing postoperative complications compared to non-smokers, with complications occurring in 27% of smokers versus 17% of non-smokers (*p* = 0.036), findings corroborated by our study [34]. Lee et al. further support these findings, demonstrating that alongside TNM staging, age, male gender, smoking, and postoperative radiotherapy are negative prognostic factors for these patients [35].

When considering the inflammatory status of the two patient groups, significant differences are observed across almost all analyzed parameters (see Table 2). Specifically, smokers exhibited significantly elevated levels of monocytes (*p* = 0.013), neutrophils (*p* = 0.001), as well as NLR (*p* = 0.025) and AISI parameters (*p* = 0.022).

In a comprehensive study conducted by Caliri et al. [36], the negative impact of smoking on the inflammatory mechanism and the host of reactive oxygen species generated by neutrophils and eosinophils, macrophages, and the imbalance thus created in overloading the antioxidant defense system and disrupting the homeostasis between oxidants and antioxidants, a disturbance known as “oxidative stress”, was presented. This imbalance leads to significant disruptions and significantly influences prognosis. International studies demonstrate the predictive capacity of elevated AISI or SIRI scores preoperatively, characteristic of patients with challenging postoperative outcomes, even with an unfavorable prognosis [28,37]. Thus, smokers expose themselves to double risks, both through the presence of oncological pathology and their smoker status.

The inflammatory response within the tumor microenvironment amplifies leukocyte proliferation, causing compromised cellular resistance and decreased T lymphocyte counts. Cytokines released in response to inflammation stimulate megakaryocytes, leading to elevated platelet levels during neoplastic progression. Thrombocytosis is detected in 39–57% of NSCLC patients and is recognized as a risk factor for metastasis [38]. Ratios such as NLR, PLR, and MLR offer valuable insights into the intricate interplay among these hematological parameters.

The patients were divided by their stage disease in three groups, (see Table 3) and data were analyzed and compared in terms of these markers. Significant changes were observed in the variation in the investigated markers across all four parameters as the disease stage increased. Specifically, increases were noted in NLR (*p* = 0.012), PLR (*p* = 0.001), SIRI (*p* = 0.01), and SII (0.015).

It is well established that the disease stage represents an absolute reference regarding the progression and prognosis of patients with pulmonary cancer. The International Association for the Staging of Lung Cancer estimates that the current 5-year survival ranges from 73% in stage IA disease to 13% in stage IV disease [39]. Therefore, the variations observed in these four aforementioned parameters confirm this aspect, emphasizing once again the predictive capacity they hold regarding the management and treatment of pulmonary cancer.

The variation in the NLR parameter revealed a statistically significant positive correlation between its value and tumor size (*p* = 0.07, r = 0.204), as well as a correlation with the Charlson index value (*p* = 0.042, r = 0.149). Thus, patients with larger tumors and multiple comorbidities also exhibited a significantly higher NLR value. An increase in tumor size typically implies a more advanced T stage [15]. Within our study, a significant increase in NLR value was observed in patients with T3-T4 compared to those with T1-T2 tumors (*p* = 0.02).

It is well established that tumor size, T stage, and N stage are negative prognostic factors for patients with oncological pathologies. Moreover, the association of advanced stages with the presence of multiple comorbidities also anticipates a less favorable prognosis [40]. However, multiple studies have brought into attention the inflammatory biomarkers that seem to adjoin the previous prognostic factors, as follows: Yin et al. demonstrated in a meta-analysis comprising 2734 cases across 14 studies that patients with elevated NLR values were predictors of poor 5-year survival [41]. Additionally, in their meta-analysis involving 3954 patients from eight studies, Li et al. revealed a significant correlation between a low LMR and decreased overall survival [42]. In a comprehensive meta-analysis across seven centers comprising 1554 patients, Qiang et al. elucidated the detrimental prognostic impact associated with elevated platelet-to-lymphocyte ratio (PLR) values, correlating with diminished 5-year survival rates [43]. Conversely, a study by Lan et al. presented a contrasting perspective, revealing expedited postoperative recovery and significantly reduced hospitalization durations in patients with low preoperative PLR and NLR values [11].

Regarding the T stage, statistically significant differences were reported between the two patient groups in terms of PLR (*p* = 0.001), AISI (*p* = 0.015), SIRI (*p* = 0.023), and SII (*p* = 0.004). Thus, the variation in these parameters is directly linked to the progression of tumor invasion, and increased values of AISI and SIRI were associated with longer hospital stays and the presence of postoperative complications following open surgeries [12,13,37].

One of the most common postoperative complications is the presence of air leaks. This complication has been associated with prolonged hospitalization and additional monitoring in ICUs. When considering patients with postoperative complications, it is observed that they come in roughly equal proportions from both urban and rural areas. However, among patients without complications, there is a predominance of those from urban areas. Due to the reluctance of rural patients toward the medical system, they typically seek hospital care when symptoms become unbearable, often at more advanced stages of the disease, thus increasing the risk of postoperative complications. Consequently, urban patients tend to seek screening and consultations more promptly in the early stages of the disease, leading to a treatment with a more favorable outcome and a lower rate of complications [44].

When discussing the predictive capacity of inflammatory status markers regarding postoperative complications (air leaks), increases in the values of all investigated parameters can be highlighted. However, although some exhibit elevated values by up to 20%, significant differences were identified in the variation in the SIRI parameter in our study (*p* = 0.041). Its ability to assess immune status prior to surgical intervention is evidenced in international studies [37], some even suggesting a potential link between a high level of this parameter and the aggressiveness of oncological progression [45]. As postoperative complications typically occur in more advanced cases, especially in smokers with multiple pathologies [46,47], we can affirm that the variation in this parameter is indeed closely related to the patient’s subsequent evolution and prognosis.

After presenting our study results and their alignment with relevant literature, it becomes evident that variations in inflammatory status parameters hold significant prognostic value for NSCLC patients. These findings underscore the intricate relationship between inflammatory markers and patient outcomes, offering valuable insights into prognostic considerations.

Moreover, the association between age, smoking, air leaks, tumor size, stage disease, Charlson index, T stage, and elevated levels of NLR, PLR, AISI, and SII highlights the multifaceted impact of these markers, enriching our understanding of their clinical significance in the postoperative period. Together, these results underscore the complex relationship between inflammatory markers and outcomes among individuals with lung cancer, offering vital insights into prognostic implications.

### Study Limitations

While our study provides valuable insights, it is crucial to acknowledge its limitations in the context of NSCLC research. Conducted retrospectively at a single medical institution, the relatively small sample size may limit the generalizability of our findings. Moreover, relying on a state-level database only captures patients undergoing surgical interventions, excluding a broader population of lung cancer cases. Additionally, the impact of changing social behaviors during the pandemic on healthcare-seeking patterns was not directly quantified. Factors such as fear of contracting COVID-19 and misinformation could have influenced the diagnosis and treatment of lung cancer, potentially affecting our results. Moreover, it is imperative to recognize the possibility of outcome misclassification, particularly concerning tumor staging (T stage and M stage). Differences in imaging methodologies and interpretations among healthcare providers could have led to misclassification, potentially affecting the perceived clinical relevance of the analyzed ratios.

Finally, it is crucial to thoroughly assess potential confounding factors, including those not addressed in our study. Additionally, exploring the fluctuating patterns of ratios before and after interventions could provide valuable insights into their clinical significance. Nevertheless, the substantial impact of this research on elucidating the role of inflammation in NSCLC highlights the necessity for ongoing investigations aimed at identifying and confirming pertinent inflammatory markers with prognostic and therapeutic implications.

## 5. Conclusions

In conclusion, this retrospective analysis reveals the advantages of some routinely determined, cost-effective, and non-invasive markers in the assessment of the potential life-threatening complications in patients undergoing lobectomies for NSCLC. Various demographic characteristics, smoking status, comorbidities, and disease stage have revealed significant associations with the preoperative inflammatory parameters in NSCLC. Consequently, systemic inflammatory markers are associated with a poorer prognosis in patients undergoing pulmonary lobectomies, indicating a correlation between inflammation, disease progression, and prognosis. Our findings underscore the importance of assessing the inflammatory status in anticipating the risk of postoperative complications and the subsequent progression of NSCLC patients. These findings are consistent with previous studies, highlighting the clinical relevance of inflammatory markers in NSCLC management.

## Figures and Tables

**Table 1:** Patients characteristics.

Characteristic	All, *n* = 187	Smokers, *n* = 66	Non-Smokers, *n* = 121	*p*-Value
Age (years, M ± SD)	60.43 ± 9.3	58.41 ± 8.73	61.54 ± 9.45	0.025
Gender, men	116 (62%)	48 (72.7%)	68 (56.2%)	0.026
Rural	71(38%)	27 (40.9%)	44 (36.4%)	0.54
Tumor location				0.054
Right lung				
Upper lobe	31 (16.6%)	17 (25.8%)	14 (11.6%)	
Middle lobe	12 (6.4%)	2 (3%)	10 (8.3%)	
Lower lobe	50 (26.7%)	20 (30.3%)	30 (24.8%)	
Left lung				
Upper lobe	51 (27.3%)	15 (22.7%)	36 (29.8%)	
Lower lobe	43 (23%)	12 (18.2%)	31 (25.6%)	
Air leak	62 (33.2%)	28 (42.2%)	34 (28.1%)	0.047
Stage				0.635
I A1	2 (1.1%)	1 (1.5%)	1 (0.8%)	
I A2	22 (11.8%)	7 (10.6%)	15 (12.4%)	
I A3	20 (10.7%)	6 (9.1%)	14 (11.6%)	
I B	33 (17.6%)	8 (12.1%)	25 (20.7%)	
II A	25 (13.4%)	11 (16.7%)	14 (11.6%)	
II B	43 (23%)	19 (28.8%)	24 (19.8%)	
III A	23 (12.3%)	6 (9.1%)	17 (14.0%)	
III B	6 (3.2%)	3 (4.5%)	3 (2.5%)	
pT				0.778
1a	4 (2.1%)	1 (1.5%)	3 (2.5%)	
1b	24 (12.8%)	8 (12.1%)	16 (13.2%)	
1c	26 (13.9%)	9 (13.6%)	17 (14%)	
2a	44 (23.5%)	11 (16.7%)	33 (27.3%)	
2b	34 (18.2%)	15 (22.7%)	19 (15.7%)	
3	29 (15.5%)	12 (18.2%)	17 (14%)	
4	13 (7%)	5 (7.6%)	8 (6.6%)	
pN				0.672
0	131 (75.3%)	45 (73.8%)	86 (76.1%)	
1	29 (16.7%)	12 (19.7%)	17 (15%)	
2	14 (8%)	4 (6.6%)	10 (8.8%)	
Tumor size (cm, M ± SD)	3.59 ± 1.85	3.93 ± 209	3.41 ± 1.69	0.105
Tumor type				0.019
Adenocarcinoma	112 (59.9%)	32 (48.5%)	80 (66.1%)	
Squamous cell carcinoma	75 (40.1%)	34 (51.5%)	41 (33.9%)	
Duration of surgery (min., M ± SD)	262.6 ± 71.36	266.58 ± 73.43	260.41 ± 71.202	0.672
Hospitalization (days, M ± SD)	14.95 ± 6.84	15.38 ± 6.91	14.72 ± 6.82	0.53
Post-surgery (days, M ± SD)	11.13 ± 5.79	11.88 ± 5.77	10.72 ± 5.79	0.192

M ± SD = mean ± standard deviation, pT = tumor invasion, pN = lymph node.

**Table 2:** Evaluated biomarkers in smoking vs. non-smoking patients.

Marker	All, *n* = 187	Smokers, *n* = 66	Non-Smokers, *n* = 121	*p*-Value
Lymphocytes	2334 ± 2023	2307 ± 672	2349 ± 2469	0.861
Monocytes	607 ± 281	675 ± 272	569 ± 280	0.013
Platelets	280,671 ± 94,565	296,424 ± 90,036	272,078 ± 96,223	0.087
Neutrophils	5627 ± 2260	6382 ± 2162	5165 ± 2203	0.001
NLR	2.53 ± 1.73	2.89 ± 1.49	2.33 ± 1.82	0.025
LMR	4.56 ± 3.69	3.92 ± 1.92	4.91 ± 4.32	0.033
PLR	144.33 ± 72.59	137.17 ± 148.24	148.24 ± 80.63	0.268
AISI	502.16± 531.22	621.85 ± 518.35	436.86 ± 528.85	0.022
SIRI	84.91 ± 58.64	90.07 ± 44.11	82.09 ± 65.22	0.322
SII	761.19 ± 702.92	883.91 ± 642.48	694.8 ± 727.79	0.07

NLR = neutrophil-to-lymphocyte ratio; LMR = lymphocyte-to-monocyte ratio; PLR = platelet-to-lymphocyte ratio; AISI = Aggregate Index of Systemic Inflammation; SIRI = Systemic Inflammation Response Index; SII = Systemic Immune Inflammation Index.

**Table 3:** Evaluated biomarkers vs. cancer stage.

Ratio	Stage I	Stage II	Stage III	*p*-Value
NLR	2.16 ± 1.29	2.75 ± 1.78	3.21 ± 2.4	0.012
PLR	123.83 ± 56.5	156.61 ± 82.76	176.57 ± 80.34	0.001
AISI	392.25 ± 352.32	639.56 ± 647.16	557.04 ± 623.76	0.02
SIRI	72.16 ± 40.79	101.98 ± 73.53	88.65 ± 57.27	0.01
SII	604.36 ± 471.99	894.67 ± 826.49	966.26 ± 897.91	0.015

NLR = neutrophil-to-lymphocyte ratio; PLR = platelet-to-lymphocyte ratio; AISI = Aggregate Index of Systemic Inflammation; SIRI = Systemic Inflammation Response Index; SII = Systemic Immune Inflammation Index.

**Table 4:** Variation in biomarkers vs. T stage.

Ratio	T1-T2	T3-T4	*p*-Value
NLR	2.26 ± 1.4	3.52 ± 2.33	0.002
PLR	135.23 ± 64.27	177.48 ± 93.28	0.001
AISI	443.87 ± 443.94	744.2 ± 731.9	0.015
SIRI	80.15 ± 55.83	106.72 ± 66.63	0.023
SII	660.19 ± 549.77	1148.8 ± 1016	0.004

NLR = neutrophil-to-lymphocyte ratio; PLR = platelet-to-lymphocyte ratio; AISI = Aggregate Index of Systemic Inflammation; SIRI = Systemic Inflammation Response Index; SII = Systemic Immune Inflammation Index

**Table 5:** Biomarkers vs. presence of air leaks.

Marker	All, n = 187	Air Leaks, *n* = 62	No Air Leaks, *n* = 125	*p*-Value
NLR	2.53 ± 1.73	2.48 ± 1.43	2.55 ± 1.87	0.795
PLR	144.33 ± 72.59	149.31 ± 66.1	141.87 ± 75.74	0.511
AISI	502.16± 531.22	570.3 ± 563.55	468.35 ± 513.4	0.218
SIRI	84.91 ± 58.64	97.31 ± 68.33	78.76 ± 52.41	0.041
SII	761.19 ± 702.92	804.02 ± 714.53	739.95 ± 699.02	0.559

NLR = neutrophil-to-lymphocyte ratio; PLR = platelet-to-lymphocyte ratio; AISI = Aggregate Index of Systemic Inflammation; SIRI = Systemic Inflammation Response Index; SII = Systemic Immune Inflammation Index.

## Data Availability

The datasets used and/or analyzed during the current study are available from the corresponding author upon reasonable request.
